# Recent summer warming in northwestern Canada exceeds the Holocene thermal maximum

**DOI:** 10.1038/s41467-019-09622-y

**Published:** 2019-04-09

**Authors:** Trevor J. Porter, Spruce W. Schoenemann, Lauren J. Davies, Eric J. Steig, Sasiri Bandara, Duane G. Froese

**Affiliations:** 1 Department of Geography, University of Toronto, Erindale Campus, Mississauga, ON Canada; 2 Environmental Sciences Department, University of Montana Western, Dillon, MT USA; 3 Department of Earth and Atmospheric Sciences, University of Alberta, Edmonton, AB Canada; 4 Department of Earth and Space Sciences, University of Washington, Seattle, WA USA

## Abstract

Eastern Beringia is one of the few Western Arctic regions where full Holocene climate reconstructions are possible. However, most full Holocene reconstructions in Eastern Beringia are based either on pollen or midges, which show conflicting early Holocene summer temperature histories. This discrepancy precludes understanding the factors that drove past (and potentially future) climate change and calls for independent proxies to advance the debate. We present a ~13.6 ka summer temperature reconstruction in central Yukon, part of Eastern Beringia, using precipitation isotopes in syngenetic permafrost. The reconstruction shows that early Holocene summers were consistently warmer than the Holocene mean, as supported by midges, and a thermal maximum at ~7.6–6.6 ka BP. This maximum was followed by a ~6 ka cooling, and later abruptly reversed by industrial-era warming leading to a modern climate that is unprecedented in the Holocene context and exceeds the Holocene thermal maximum by +1.7 ± 0.7 °C.

## Abstract (web summary)

Traditional precipitation isotope archives (e.g., ice cores) are fundamental to our knowledge of past climate but limited to glaciated locales. Here the authors show that pore ice in relict permafrost holds equal promise as a proxy and use it to provide insights on the Holocene summer climate history of northwestern Canada.

## Introduction

The Arctic has warmed faster than any other region this past century, owing to climate-cryosphere feedbacks^1^. This warming is driving a range of biotic and abiotic changes that directly impact northern communities and ecosystems^2,3^, as well as enhanced C fluxes to the atmosphere from thawing permafrost^4^ and ice loss from glaciers and ice caps^5^, which have implications for global climate and sea level, respectively. The broad-reaching effects of Arctic change highlight the need to advance our knowledge of the Arctic system.

Current knowledge of the Arctic system is informed by a sparse network of relatively short (~50–100 years) instrumental records. However, the Arctic is transitioning to a warmer state that appears to have no analogue in the historical record. Natural proxy archives (e.g. ice cores and lake sediments) offer an alternative source of data on past climate and environmental change extending back thousands of years, with analogues from the distant past that may be especially relevant for anticipating the form of a future, warmer Arctic^6^. Arctic proxy reconstructions for the Common Era (CE, last 2 ka)^7^ and Holocene (last ~11.7 ka)^8,9^ broadly agree that the late Holocene was characterised by a long cooling trend that ended with abrupt warming during the industrial era. Many proxy reconstructions agree that industrial-era warming was exceptional in overall rate, but not magnitude in the Holocene context^10,11^, while others suggest that industrial-era warming has been exceptional in both regards^6,12,13^. Differences can be explained by spatial heterogeneity in the climate field or proxy-related noise (e.g. chronological error, secondary climate sensitivities and differences in seasonality). Noise is inherent to all proxies, but recovery of the climate signal can be optimised through replication and a diverse, multi-proxy approach.

Eastern Beringia, the contiguous area of Yukon and Alaska that was ice free during the last glaciation, is one Arctic borderland where the Holocene climate history is still debated. A review by Kaufman et al.^9^ showed that nearly all quantitative, summer-specific temperature reconstructions from Eastern Beringia with early Holocene coverage are based on two proxy types—pollen and midges (Chironomidae) in lake sediments. Regionally, pollen and midges agree there was a climate optimum at ~7–5 ka BP, followed by a long-term cooling. However, these proxies show large differences before 8 ka BP with pollen indicating that early Holocene summers were persistently colder than the Holocene mean (Δ*T* range = –3.3 to –0.5 °C; mean Δ*T* = –1.6 °C), and midges showing that early Holocene summers were similar to the Holocene mean (Δ*T* range = –0.1 to + 0.4 °C; mean Δ*T* = + 0.1 °C). This discrepancy confounds our understanding of the drivers of early Holocene climate change and highlights the need for independent proxies to resolve this debate.

Precipitation isotope ratios—^2^H/^1^H (δD_precip_) and ^18^O/^16^O (δ^18^O_precip_)—in ice core records have a long history of use as a temperature proxy^14–16^ and have played a crucial role in shaping our knowledge of Quaternary climates in glaciated locales. More recently, this concept has been applied to Pleistocene and Holocene ice wedges (snowmelt derived) in permafrost environments in parts of Siberia^12,17^ and Eastern Beringia^18–20^ to reconstruct winter δ_precip_ (δD_precip_ or δ^18^O_precip_) and temperature. Relict pore ice in syngenetic (aggrading) permafrost also shows potential as a δ_precip_ proxy^19^, although there are few pore ice studies with a paleoclimate focus, and only one example from Siberia^21^ where a Holocene δ_pore ice_ series was developed and used for qualitative (warmer vs. cooler) temperature inferences.

**Fig. 1:**
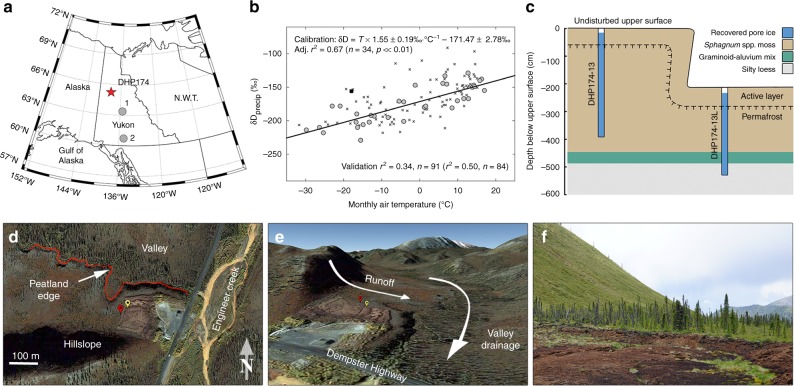
Study region and DHP174 (Dempster Highway Peatland, near 174 km marker) site. **a** Regional map showing the DHP174 site (red star) and GNIP (Global Network of Isotopes in Precipitation) sites (1) Mayo and (2) Whitehorse; **b** the local δD_precip_-temperature regression line calibrated with Mayo GNIP data (circles; excluding any leverage points marked with a square, see Methods); the validation *r*^2^ reflects the performance of the calibration model in predicting Whitehorse GNIP δD_precip_ (crosses); the validation *r*^2^ in brackets excludes extreme outlier residuals >2.5× the interquartile range (see Supplementary Fig. 1 for δ^18^O_precip_-T line); **c** lithostratigraphy and position of the two core profiles (~50 m lateral distance); **d** overhead and **e** DEM oblique view of the study site—Google Earth and DigitalGlobe satellite images—with points marking the upper (red) and lower (yellow) core site locations; and **f** ground-level photograph from the peatland edge looking southwest across the disturbed surface

In this study, we present the first full Holocene, quantitative temperature reconstruction from a sequence of relict pore ice in syngenetic permafrost from a soligenous peatland called DHP174 (Dempster Highway Peatland, near 174 km marker) in central Yukon (Fig. 1a), part of Eastern Beringia. Surface peat accumulation is the main driver of permafrost aggradation at this site, which simultaneously archives a warm-season blend of precipitation (integrated as pore water) that reaches the base of the seasonally thawed active layer in the summer. This reconstruction is enabled by a significant positive association between local air temperatures and precipitation isotope ratios (Fig. 1b; Supplementary Fig. 1). Our pore ice record is conceptually similar to an ice core record in that it preserves a stratigraphic profile of ancient precipitation, but distinct because of its seasonal bias toward summer precipitation and a fractionation that occurs during freezing and causes relict pore ice to be enriched in δD and δ^18^O relative to the initial pore waters^22^. Based on this record, we develop a full Holocene δD_precip (pore ice)_-based summer Δ*T* reconstruction, and an abiotic perspective to advance our understanding of early Holocene climate in Eastern Beringia.

## Results

### Paleoenvironment and stratigraphy

The DHP174 peat-sediment composite record (5.32 m composite depth) is the product of two core profiles that overlap stratigraphically and includes three lithostratigraphic units (Fig. 1c) that were deposited over the last ~15.6 ka (see Methods). Unit 1 (5.32–4.78 m) is silty loess and was deposited between 15.6 and 14.3 ka cal BP. Unit 2 (4.78–4.38 m) is a graminoid-rich silty unit that aggraded relatively slowly (~0.1 cm decade^−1^ average) from 14.3 to 11.2 ka cal BP. Unit 2 overlaps with the Younger Dryas interval (~12.9–11.7 ka cal BP), when the climate of northern Alaska was much colder than today^18,23^. Finally, Unit 3 (4.38 m to the surface, 10.5 ka cal BP to present) is primarily *Sphagnum* moss, reflecting an aggrading peatland phase. The peat sequence accumulated at a relatively stable rate of ~0.4 cm decade^−1^, with the exception of higher rates at the time of peatland inception (4.38–4.13 m, ~2 cm decade^−1^) and a brief slowdown near the core top (0.21–0.15 m, ~0.1 cm decade^−1^) during the period ~1000–200 cal yr BP.

Peat fibres were exceptionally well preserved throughout the profile (Supplementary Fig. 2a–c), which suggests a relatively uninterrupted aggradation of permafrost since inception, and a syngenetic origin of relict pore ice in this sequence. Downcore ice content is primarily structureless pore ice (Supplementary Fig. 2) representing pore waters that froze in situ^24^. Rare ice lenses (typically <0.5 cm thick; Supplementary Fig. 2b) were encountered in Unit 1 (*n* = 7) and Unit 3 (*n* = 2) but were not sampled. Ice lenses represent active layer waters drawn to the freezing front by cryosuction^24^, a process that is common in fine-grained sediments and may explain their higher number in Unit 1. Pore ice and lens ice derive from potentially different active layer water and cannot be directly compared. In this study, we focus exclusively on the dominant form of ice—relict pore ice.

In contrast to the ~15.6-ka-long peat-sediment chronology described above, the relict pore ice hosted in the same peat-sediment sequence spans the last ~13.6 ka cal BP. The shorter relict pore ice chronology relates to the maximum depth at which active layer pore ice forms and becomes ‘relict’, which equals the maximum active layer thickness (ALT_max_). ALT_max_ is defined as the thickest active layer likely to occur in a given climatic period and is ~58 cm for the study site today (Supplementary Note 1). Because relict pore ice forms below the surface it will always be younger than the host sediments. For example, ca. modern relict ice (–68 cal yr BP; 58 cm below ground) is hosted in fossil peats that grew at the surface ~2065 cal yr BP (i.e. the depositional age of the peats). Relict pore ice in the basal sediments is likewise ~2 ka younger than the depositional age of the hosting sediments (see Methods for full details on the relict pore ice chronology).

### Composite relict pore ice record

Relict pore ice δD and δ^18^O series from the upper and lower core profiles are compared and combined in a composite series in this section. For the upper core, relict pore ice includes all pore ice from depths greater than ALT_max_ or ~58 cm (i.e. excluding all active layer pore ice). For the lower core profile, we consider all pore ice at or below a composite depth of 290 cm (~90 cm below the lower coring surface) to be relict pore ice. The deeper cut-off depth to relict pore ice in the lower core avoids pore ice that has been overprinted by modern waters following disturbance of the lower surface (Supplementary Fig. 3).

**Fig. 2:**
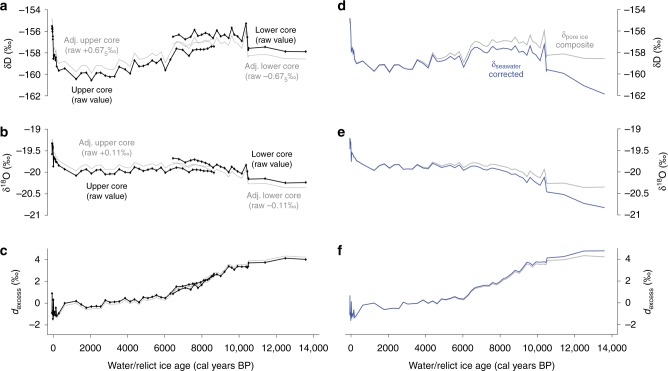
Holocene pore ice record. **a**–**c** Comparison of raw δD, δ^18^O and *d*_excess_ pore ice series (black lines) from the upper vs. lower cores, and ‘adjusted’ δD, δ^18^O and *d*_excess_ series (grey lines), which are each corrected by half of the mean offsets in δD and δ^18^O between the upper and lower cores during the period of overlap; **d**–**f** Composite pore ice δD, δ^18^O and *d*_excess_ (grey lines), and δ_seawater_-corrected pore ice records (blue lines)

The upper and lower relict pore ice records collectively span the last ~13.6 ka cal BP. The two records show small systematic differences in their mean δD and δ^18^O values during the period of overlap from 8.5 to 6.4 ka cal BP (composite depths 380–290 cm), the upper core being relatively depleted compared to the lower core in δD and δ^18^O (Fig. 2a, b). There is no offset in *d*_excess_ (*d*_excess_ = δD – 8 × δ^18^O) (Fig. 2c) The δD and δ^18^O differences are small in absolute terms (the mean offsets are 1.35‰ and 0.22‰ for δD and δ^18^O, respectively) and are not unexpected for different sites across a landscape given the same source water. As we discuss below, relict pore ice is enriched in heavy isotopologues relative to the initial pore waters due a fractionation that occurs during the freezing process^22^. The overall enrichment (*ε*_ice-water_) depends on the rates of freezing and molecular diffusivity. For our site, the lower active layer is saturated in the late summer and, thus, the rate of freezing will be buffered by latent heat effects^25^. However, water table depth and total latent heat content are unlikely to be spatially homogenous due to variations in microtopography and slope, and therefore the rate of freezing and *ε*_ice-water_ are also unlikely to be homogenous. Because the two core locations (~50 m apart) receive the same precipitation and runoff, spatial variability in *ε*_ice-water_ is a probable explanation for the small offsets in δ_pore ice_.

Regardless of origin, the offsets are systematic and the cores show coherent variations at 10^2^–10^3^ year timescales (Fig. 2a, b), which suggests that both cores are in fact archiving the same source water. To enable a direct comparison of the two series in a composite record, we adjusted the upper core δD_pore ice_ and δ^18^O_pore ice_ series by adding a constant equal to half of the mean δD and δ^18^O offsets, and the lower core δD_pore ice_ and δ^18^O_pore ice_ by subtracting half of the mean δD and δ^18^O offsets (Fig. 2a, b). This aligns both series to a common mean value during their period of overlap without modifying the individual trends, and, thus, the composite δD_pore ice_ and δ^18^O_pore ice_ records (Fig. 2d, e) also accurately depict the raw δ_pore ice_ trends. These adjustments are small in absolute terms and do not influence trends in *d*_excess_ (Fig. 2c–f) nor conclusions on seasonality of relict ice, which we based on raw δ_pore ice_ (next section).

The composite record covers the last ~13.6 ka with an average time-step of ~158 years between pore ice samples since 10.5 ka cal BP; however, from 13.6–10.5 ka cal BP, the average time-step is relatively coarse (~1020 years between samples) due to low accumulation rates just prior to the peatland initiation.

### δ_pore ice_ systematics and seasonality

**Fig. 3:**
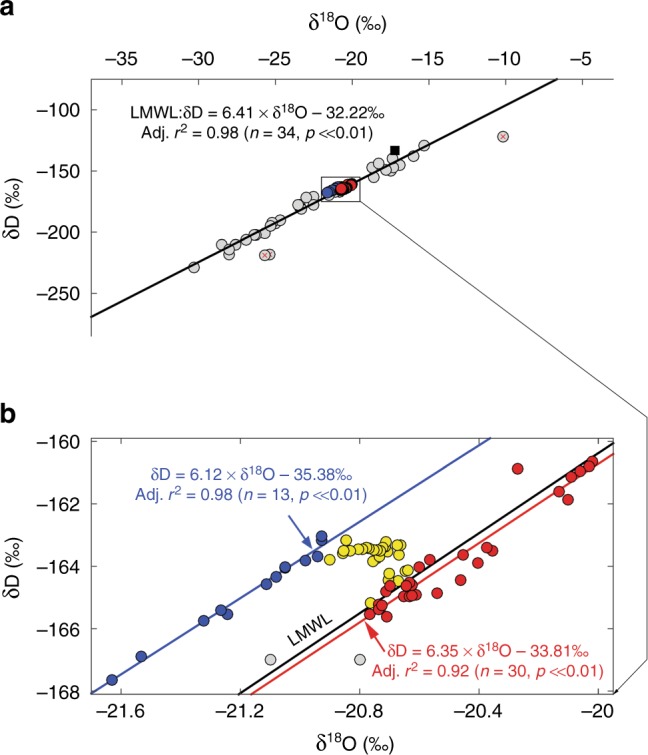
Co-isotope plot of local GNIP (Global Network of Isotopes in Precipitation) data and δ_pore ice_. **a** Mayo GNIP data (grey circles) used to constrain the Local Meteoric Water Line (LMWL; excluding any leverage points marked with a square and likely evaporation-enriched data points marked with red crosses) and the composite DHP174 (Dempster Highway Peatland, near 174 km marker) δ_pore ice_ record (δ_seawater_-corrected—as in Fig. 2d, e); δ_pore ice_ data points are colour coded by age classes >9.2 ka BP (blue circles), 9.2–5 ka BP (yellow circles) and <5  ka BP (red circles); **b** enlarged inset (as shown in subplot ‘a’) with separate regression lines for the LMWL (black), and early Holocene (>9.2 ka cal BP; blue) and late Holocene (<5 ka cal BP; red) δ_pore ice_

Co-isotope (δD and δ^18^O) measurements of relict pore ice plot tightly on the Local Meteoric Water Line (LMWL; Fig. 3a), indicating that the pore ice is derived from meteoric waters that froze in situ rather than evaporatively enriched soil waters, which would plot below the LMWL along a shallow evaporation line^26^. However, relict pore ice older than 5 ka BP plots above the modern LMWL (Fig. 3b), which may reflect changing Holocene boundary conditions as we discuss in the next section. The remainder of this section focuses on seasonality of ca. modern (top-of-permafrost) δ_pore ice_.

Relict pore ice is ‘first ice’ (the first ice to form in a bottom-up or top-down freezeback sequence) that once formed at the bottom of a paleo-active layer. First ice is enriched in heavy isotopes relative to the initial pore waters^22^. Based on an analysis of DHP174-13 active layer ice, the enrichment is ~5.8‰ for δD and ~0.8‰ for δ^18^O, and is not expected to vary significantly through time due to complacent freezeback conditions in a saturated active layer and unchanging peat lithology for most of the record (Supplementary Note 2). To establish the δ_precip_ seasonality of ca. modern δ_pore ice_, we account for these enrichments to estimate the initial δ_pore water_, which can be compared to the δ_precip_ climatology of the nearest GNIP site at Mayo, Yukon.

**Fig. 4:**
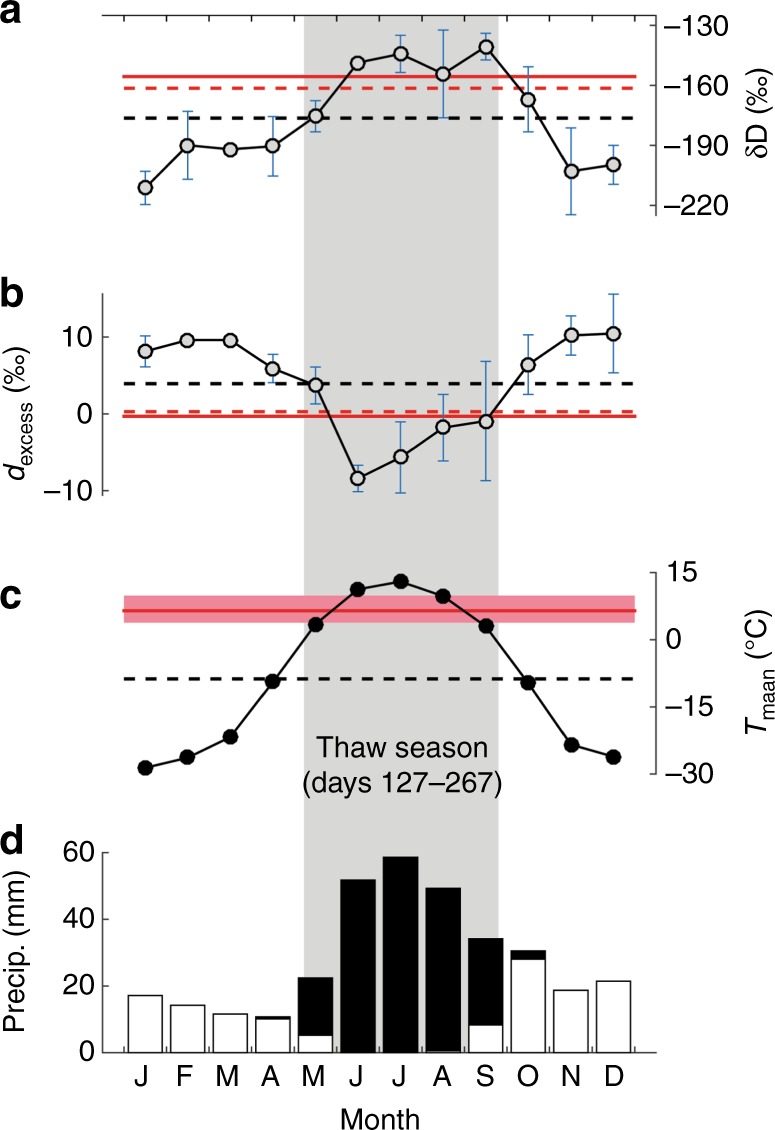
Precipitation and pore ice isotope climatology. **a** Monthly δD_precip_ (see Supplementary Fig. 4 for δ^18^O) and **b** monthly *d*_excess_ calculated from Mayo GNIP (Global Network of Isotopes in Precipitation) data (grey circles and 1*σ* error bars; excludes suspected evaporation-enriched data points shown in Fig. 3a); Mayo mean annual δD_precip_ and *d*_excess_ (dashed black lines), mean DHP174-13 (Dempster Highway Peatland, near 174 km marker) top-of-permafrost (58–60 cm) pore ice δD and *d*_excess_ (solid red lines) and estimated initial pore water δD and *d*_excess_ (dashed red lines) are indicated; **c** comparison of δD_pore ice (top-of-permafrost)_-based temperature estimate (red line; red band = ± 1*σ* uncertainty; Methods) and mean monthly (black circles) and mean annual (black dashed line) temperatures at the Ogilvie River Environment Canada station (1971–2007 period of record; Supplementary Note 3); **d** mean total monthly snowfall (white bars; in snow-water equivalent) and rain (black bars) at Ogilvie River. The shaded area represents the average thaw season (i.e. period when mean daily air temperatures are above freezing)

Mean δD_pore water_ and δ^18^O_pore water_ estimates for the uppermost 3 cm of DHP174-13 relict pore ice (58–61 cm; representing the last ~3 decades of syngenetic permafrost) are −161.5 and −20.2‰, respectively, with a mean *d*_excess_ value of +0.1‰. Compared to local meteoric waters from Mayo, our δD_pore water_ estimate is enriched relative to mean annual δD_precip_ and within range of thaw season (May–September) δD_precip_ values (Fig. 4a). The pore water *d*_excess_ estimate is also biased toward warm-season precipitation values (i.e. depleted relative to annual precipitation *d*_excess_ and enriched compared to the warmest months; Fig. 4b). Both comparisons suggest relict pore ice derives mainly from warm-season precipitation. However, a caveat of these comparisons is that the temporal integrations of the Mayo δD_precip_ record (1985–1989, variable replication depending on month) and ca. modern pore ice (last ~3 decades) are unequal, which may impact how closely they agree. Mayo δD_precip_ data generally follow expectations of more depleted values during cold winter months and enriched values during warm summer months (as in Fig. 1b), but discrepancies do occur (e.g., peak δD_precip_ in September, which is not the warmest month), which are likely attributable to the limited replication in the Mayo record. The proposed seasonality of relict pore ice can also be corroborated by comparing δ_pore ice_-based temperature estimates (Methods) with the local climatology as constrained by climate data from the Ogilvie River station (Supplementary Note 3), ~17 km north of the DHP174 site. The average temperature estimate is 6.3 ± 3.4 °C (1*σ*) based on δD_pore ice_ (Fig. 4c), and 7.0 ± 3.3 °C (1*σ*) based on δ^18^O_pore ice_ (Supplementary Fig. 4c), which are the same within error. Both estimates are bracketed by mean May–September temperatures in the study area and provide further confirmation that relict pore ice derives primarily from warm-season precipitation.

A warm-season seasonality is also expected for climatological and hydrological reasons. Roughly 65% of the annual precipitation budget is delivered during the thaw season, the bulk of which is rain (Fig. 4d). Secondly, the typical active layer hydrology style of raised northern peat plateaus favours a warm-season seasonality. At the time of snowmelt, the active layer is largely undeveloped (e.g. 0–10 cm), which forces meltwaters to drain near the surface where saturated hydraulic conductivity (*K*_s_) is high (10–1000 m day^−1^, 0–10 cm depth)^27^. Conversely, as the active layer thaws in the summer, precipitation percolates to greater depths and will drain slowly due to lower *K*_s_ in the deeper active layer (0.5–5 m day^−1^ below 20 cm)^27^. This hydrological style favours greater representation of warm-season precipitation in the lower active layer, where pore waters eventually freeze and become relict following permafrost aggradation.

Finally, we note that the composite record of pore ice (Fig. 2d, e) is characterised by relatively small inter-sample δ_pore ice_ variability (on average 0.4‰ for δD and 0.1‰ for δ^18^O) in comparison to the large inter-annual (weather-related) variability in δ_precip_ that is evident in local GNIP records for most months (see δD_precip_ ± 1*σ*; Fig. 4a). The absence of extreme variability in δ_pore ice_ suggests that each pore ice sample represents a multi-year seasonal blend of precipitation, whereby weather-related fluctuations in δ_precip_ are attenuated through the mixing of precipitation from several warm-season months, and multiple years facilitated by repeated freeze–thaw cycles. This is logical given the slow peatland aggradation rate (~0.4 cm decade^−1^), which dictates that up to ~20–30 years of mostly warm-season precipitation can mix in the lower active layer before the permafrost table aggrades 1 cm, thereby trapping pore ice situated at the former permafrost table as constrained by ALT_max_. While ALT_max_ will not be reached every thaw season (Supplementary Note 1), it is the infrequent, warmer-than-normal summers that can reanimate and mix with pore ice that froze at intermediate thaw depths (<ALT_max_) during prior, cooler summers. Thus, natural averaging of multi-year pore ice in the active layer ensures a climatic timescale integration (depending on the aggradation rate) of δ_precip_ and representation of cool and warm summers in any given sample of δ_pore ice_, which explains the lack of extreme inter-sample δ_pore ice_ variability noted above.

### Holocene trends in δD, δ^18^O and *d*_excess_

Over Holocene and longer timescales, the isotopic composition of seawater is variable. The composite δ_pore ice_ records were corrected for changes in δ_seawater_ (Fig. 2d–f; see Methods) in order to isolate δ_pore ice_ variability related to changes in climate. The δD_pore ice (seawater corrected)_ record (Fig. 2d) shows a rising trend through the Pleistocene–Holocene transition reaching some of the most enriched values of the early Holocene at ~10 ka cal BP, then a lesser but still positive trend between 10 and 6.6 ka cal BP, followed by an ~6-ka-long negative trend leading to the most depleted values on record in the early CE (~2 ka BP), and last an abrupt trend reversal that began ~150 yr BP and has culminated in the most enriched δD_pore ice_ values at present. It is possible this reversal started before 150 yr BP, but the 1000–150 yr BP portion of our chronology is not well resolved due to the slow peat accumulation during this period, as discussed above. The reason for the slow accumulation is not known but could reflect a local climate limitation to peat growth (e.g. more arid) during the late pre-industrial era.

The δ^18^O_pore ice (seawater corrected)_ record (Fig. 2e) documents similar low- and high-frequency trends, but a more pronounced positive trend during the early Holocene between ~9.2 and 5 ka cal BP. This difference is reflected in the *d*_excess_ record (Fig. 2f), which transitions from high mean values (~4 ‰) at 9.2 ka cal BP to low mean values (~0‰) by 5 ka cal BP. *d*_excess_ did not change much since the mid-Holocene. Under today’s δ_precip_ boundary conditions (i.e. as defined by the present-day LMWL), *d*_excess_ values >4‰ are common during the cold-season months (October–April), and so the *d*_excess_ change in our pore ice record could be interpreted as a change in the precipitation seasonality (e.g. ratio of warm-to-cold-season precipitation) integrated in pore ice; however, this interpretation would also require a constant LMWL, which the co-isotope results (Fig. 3b) show is an invalid assumption. A precipitation seasonality mechanism is also unlikely given the active layer hydrology of northern peat plateaus, as discussed above, which precludes snowmelt from the deep active layer where relict pore ice is created. The Holocene *d*_excess_ trend is attributed to a transition from a paleo-LMWL (with a more positive intercept compared to today) defined by early Holocene relict pore ice (>~9.2 ka cal BP) to the contemporary LWML defined by modern GNIP data, but which aptly describes late Holocene relict pore ice younger than 5  ka BP (Fig. 3b). Continental boundary conditions such as local orographic effects and mean weather trajectories define the slope of the LWML^22^, whereas the intercept of the LMWL (which points to the *d*_excess_ of the first evaporate) is influenced by the mean evaporative boundary conditions at the moisture source^28^. Given the similar slopes but different intercepts of the paleo vs. modern LMWLs, we conclude that a change in evaporative boundary conditions at the moisture source is the most likely explanation for the Holocene *d*_excess_ transition.

The *d*_excess_ parameter reflects non-equilibrium partitioning of HDO relative to the slower moving H_2_^18^O isotopologue in the evaporate leaving the ocean. *d*_excess_ is sensitive to factors that influence the evaporation rate including sea surface temperature (SST) and relative humidity (RH)^28^, which has made it a useful proxy in ice core studies for reconstructing changes that are far removed from the ice core site^14^. The North Pacific is the primary moisture source for central Yukon today^19^. Assuming a stable moisture source through the Holocene, the observed *d*_excess_ trend could reflect a cooling in North Pacific SST and/or rise in RH in the moisture source region. SST proxies encompassing a large area of the North Pacific broadly agree that SSTs peaked around ~10 ka and cooled by ~1–2 K by the mid-Holocene^29,30^. However, spatial coherence diminishes in the late Holocene with some SST reconstructions, indicating warming and others cooling. The balance of paleo-SST evidence is inconclusive, but hints at a possible and spatially complex relation between *d*_excess_ and SST.

Alternatively, the *d*_excess_ trend could indicate a geographic shift in the moisture source region with potentially different mean SST or RH at the different localities. The initial timing of the *d*_excess_ trend is coincident with the final collapse of the Laurentide ice sheet (LIS) from ~11–7 ka BP^31^. The LIS strongly impacted atmospheric circulation and climate in western Eurasia^32^. Likewise, climate modelling studies suggest that the collapse of the LIS had a warming effect on E. Beringia^33^, although the dynamical response of summertime atmospheric circulation patterns in the North Pacific sector to LIS collapse is unknown.

The co-isotope results indicate a transition from one set of δ_precip_ boundary conditions to another. Although the physical mechanisms behind this transition are not well known, further interrogation of possible changes—such as modified SST or RH at a constant moisture source, or a geographic change in moisture source (e.g. due to altered atmospheric circulation pattern in the summer months)—will benefit from greater replication of the paleo-SST field and isotope-enabled GCM (General Circulation Model) experiments.

### Summer temperature reconstruction

Under modern δ_precip_ boundary conditions, δD_precip_ and δ^18^O_precip_ are both temperature-sensitive proxies (Fig. 1b; Supplementary Fig. 1). However, if the modern δ_precip_-temperature transfer functions (both of which are linear) were applied to the δD_pore ice_ and δ^18^O_pore ice_ records, they would yield different reconstructions given their contrasting trends (Fig. 2d, e)—and clearly both cannot be correct. As evidenced by the early to late Holocene LMWL transition (as discussed above), δ_precip_ boundary conditions did change and were likely driven by changes in evaporative conditions at the moisture source. The slow-moving H_2_^18^O isotopologue is especially sensitive to non-equilibrium effects driven by changes in evaporation rate^28^. By comparison, HDO is relatively insensitive to this effect. For that reason, we consider δD_pore ice_ to be a potentially more reliable local temperature proxy and use it as the basis for our Δ*T* anomaly reconstruction (see Methods).

**Fig. 5:**
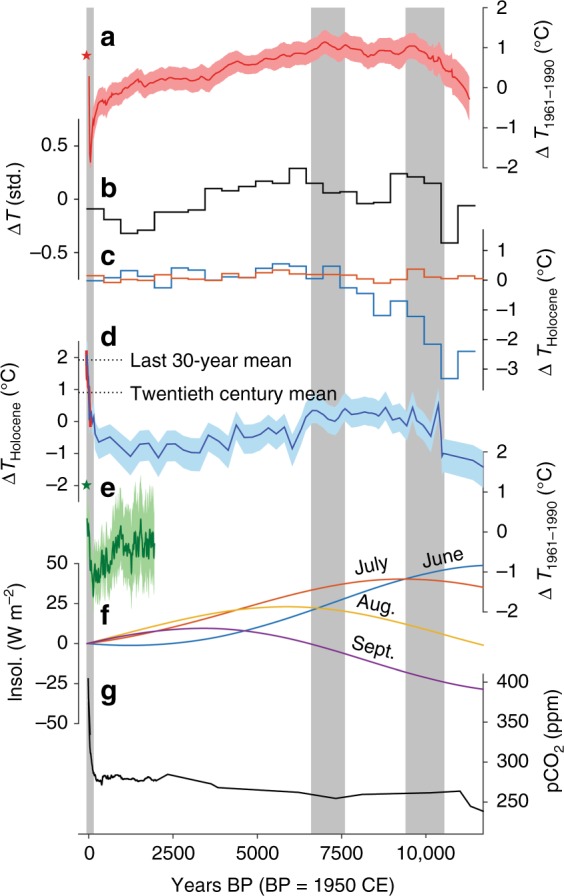
Δ*T* reconstructions and climate forcings. **a** Northern extratropical (30–90°N) multi-proxy Δ*T* reconstruction^11^ (red; filled area = ±1*σ* uncertainty) and mean 1997–2016 GISTEMP^37^ annual Δ*T* for the same area (red star); **b** E. Beringia multi-proxy ‘all-temperature’ composite^9^; **c** E. Beringia composite pollen- (blue) and midge-based (orange) summer Δ*T* reconstructions^9^; **d** DHP174 (Dempster Highway Peatland, near 174 km marker) δD_pore ice_-based summer Δ*T* reconstruction (blue; filled area = ±1*σ*; this study) and smoothed 1900–2017 May–September GISTEMP Δ*T* anomalies for the study area (red; n.b., GISTEMP anomalies were smoothed with a 40-year cubic spline and normalised to the twentieth century mean of the δD_pore ice_ Δ*T* reconstruction; a 1991–2001 data gap in GISTEMP was filled with ERA-interim data^64^); **e** 40-year smoothed Arctic (60–90°N) multi-proxy Δ*T* reconstruction^7^ (green; filled area = ±1*σ*) and mean 1997–2016 GISTEMP^37^ annual Δ*T* for the same area (green star); **f** June–September insolation at 65°N^65^; **g** Vostok, Law Dome and Mauna Loa composite *p*CO_2_ record^66–68^. Grey bars highlight warming intervals in the δD_pore ice_-based Δ*T* reconstruction that are also evident in other Δ*T* reconstructions in E. Beringia and at broader spatial scales

The Δ*T* reconstruction (expressed as anomalies w.r.t. Holocene mean; Fig. 5d) shows a strong warming trend during the Pleistocene–Holocene transition, and variable Δ*T* anomalies ranging from –0.5 to +0.5 °C during the early Holocene (10.4–9.6 ka cal BP) with a mean Δ*T* of +0.1 °C. Local summer Δ*T* reconstructions from pollen and midges show major differences during this interval, with pollen indicating cool Δ*T* values from –2.2 to –1.2 °C, and midges indicating warm values from +0.1 to +0.4 °C (Fig. 5c). This difference may be due to limitations of the modern analogue technique (MAT), which is the quantitative method used to estimate summer temperatures from the E. Beringian pollen records. As discussed by Kaufman et al.^9^, it is notable that qualitative interpretations based on pollen in this region have reached the opposite conclusion that the early Holocene was warm. A warm climate is also indicated by a diverse suite of qualitative proxy evidence in E. Beringia, including high frequencies of thaw lake and peatland initiation^9^; a multi-proxy ‘all-temperature’ composite record that depicts the dominant mode of variability of all-temperature-sensitive marine and terrestrial records (Fig. 5b); and warmer SSTs as discussed above. However, not all of these supporting proxies are necessarily sensitive to summer temperatures exclusively. Last, a brief thermal maximum at ~10 ka cal BP is also evident in a multi-proxy temperature record representing the northern extratropics (30–90°N) (Fig. 5a), suggesting this phase of early Holocene warmth was widespread. The balance of evidence favours a warm early Holocene. It remains unclear why MAT-based interpretations of pollen in this region, on average, suggest colder summers. Further studies are needed to understand this discrepancy, especially given the popular use of pollen data in Quaternary studies.

From 9.6 to 6.6 ka cal BP, our reconstruction shows a sustained warming with Δ*T* anomalies ranging from –0.1 to +0.4 °C, and an average Δ*T* of 0.2 °C (Fig. 5d). Local maxima in Δ*T* occur at 9.6 ka cal BP (+0.4 °C), 8.8 ka cal BP (+0.4 °C), 7.6 ka cal BP (+0.4 °C) and 6.8–6.6 ka cal BP (+0.3 °C). Individual Δ*T* anomalies should not be over-interpreted; however, it is notable that the +0.4 °C anomaly at 8.8  ka cal BP coincides with a deep thaw unconformity preserved in permafrost near Mayo, which is dated to 8.87 ± 0.2 ka BP and has been linked to a brief period of exceptionally warm conditions^34^. We also note that the later warm interval between 7.6 and 6.6 ka cal BP coincides with a period of Arctic treeline expansion in the Mackenzie Delta region (~400 km N.E. of DHP174) that began ~8 ka BP^35^. The 7.6–6.6 ka cal BP warm interval also matches a thermal maximum in the extratropical composite (Fig. 5a). The E. Beringia ‘all-temperature’ composite shows a slightly later thermal maximum at ~6.5–6.0 ka BP (Fig. 5b). The regional pollen composite shows warm summers (+0.47 °C) from 7.5 to 7.0 ka BP (comparable to the pore ice Δ*T* anomaly at ~7.6 ka BP), but slightly warmer summers (+0.55 °C) from 6.0 to 5.5 ka BP (Fig. 5c). Similarly, the midge composite shows warm summers (+0.19 °C) from 7.5 to 6.5 ka BP, but even warmer summers (+0.34 °C) from 6.0 to 5.5 ka BP. Differences in magnitude and timing of early-middle Holocene peak warmth between the summer-specific proxies (i.e. pollen, midges and δD_pore ice_) are relatively small, and to some extent are likely explained by the unequal spatial representation of the different records. Additional pore ice records across the region may improve inter-proxy coherence.

From 6.6 to 1.2 ka cal BP, our reconstruction shows a long-term cooling trend of –0.16 °C ka^−1^ (*p* = 6.82 × 10^–5^) (Fig. 5d). Prominent cooling trends over the same interval are documented in the E. Beringia multi-proxy composite and extratropical multi-proxy composite (Fig. 5a, b). The extratropical composite shows a cooling trend of the same magnitude (–0.17 °C ka^−1^) between 6.6 and 1.2 ka BP. Regional pollen and midge composites also show cooling trends through this interval, but at lesser rates of change (–0.04 and –0.05 °C ka^−1^, respectively; Fig. 5c). Differences in cooling rates between the records may be explained by seasonality or spatial (e.g. continentality) inequities.

From ~1200 to 150 cal yr BP our reconstruction shows a gradual warming trend. A similar warming trend over the last ~1–2 ka is observed in the E. Beringia all-temperature composite (Fig. 5b). However, a warming trend during this interval is relatively uncommon to CE temperature reconstructions globally^36^, and is not supported by the multi-proxy composites from 30 to 90°N (Fig. 5a) and 60 to 90°N (Fig. 5e), which show a cooling trend during this time. Due to the slow peat accumulation rates from ~1000 to 150 cal yr BP (~0.1 cm ka^−1^), the late pre-industrial era is not well resolved in our pore ice record; therefore, our reconstruction should not be used for detailed estimates of temperature change during this interval.

Finally, our reconstruction shows accelerated warming since ~150 cal yr BP, culminating in the warmest summer climate at present, which (based on the last ~1 decade of relict pore ice, or the uppermost 1 cm of relict pore ice) we estimate is 2.1 ± 0.7 °C warmer than the Holocene average or 1.7 ± 0.7 °C warmer than the peak Δ*T* anomalies during the early-middle Holocene thermal maximum (Fig. 5d). The average ∆*T* anomaly for the last ~30 years of relict pore ice (uppermost 5 cm of relict pore ice) is +1.9 ± 0.7 °C warmer than the Holocene mean. The last ~100 years of our record is especially well resolved (e.g. sub-decadal for the top 5 cm of relict pore ice) due to the finer pore ice sampling resolution through this interval, and the historic cryptotephra (1912 CE Novarupta-Katmai at 10 cm depth) and post-bomb ^14^C dating (−35 cal yr BP at 4–5 cm depth) that constrain this part of the chronology (Tables S1, S2). Our reconstruction shows ~2 °C of warming over the last 100 years, which matches the overall increase in observed May–September temperature anomalies from this region^37^ (Fig. 5d). Similar warming rates are evident in the extratropical and Arctic composites (Fig. 5a–e), and temperature estimates from the Agassiz ice core ‘melt’ record, Ellesmere Island^10^. However, the magnitude of warming in the industrial era vs. early Holocene is still debated. The extratropical multi-proxy composite suggests the modern climate has not yet exceeded early Holocene peak warmth^11^, while some land-only (North America and Europe) pollen-based temperature reconstructions suggest the last decade was ~1.2 °C warmer than the Holocene mean^13^. Retreating glaciers on Baffin Island in the Canadian Arctic have exposed rooted plant fossils with non-finite ^14^C ages, which indicates that recent climate warming at that locality is unprecedented since the last interglacial^6^. While these select examples do not resolve the debate over whether today’s climate is exceptional everywhere in the Holocene context, they do support the idea that the magnitude of early Holocene warming has varied spatially. At our site in easternmost Beringia, the present-day summer climate does appear to be exceptional in the last 13.6 ka cal BP, and our reconstruction suggests that summer temperatures in recent decades have consistently exceeded the Holocene Thermal Maximum since about the mid-20th century.

Variability in Holocene summer climate in this region owes to multiple factors. The centroid of the July–August insolation curves and subsequent decline is closely aligned with the low-frequency trend in our reconstruction (Fig. 5d–f), and implies that summer insolation played an important role in pacing summer temperatures. The importance of insolation in driving Holocene climate in this region is corroborated by numerical climate model simulations^33^. However, other factors likely had a moderating effect. For example, the flooding of the Bering Strait (as late as 11 ka BP^38^) and development of thaw lakes in Yukon and Alaska in the early–middle Holocene probably had a regional cooling effect on summer climate^33^, while the LIS collapse and increases in shrub and boreal taxa likely had a warming effect^33,39^. The integration of all relevant forcings and feedbacks likely explains some of the more complex variabilities in early Holocene climate that diverge from a simple insolation-based interpretation. Finally, the industrial-era warming in our record (~2 °C warming over the last century) is coeval with the rapid increase in global *p*CO_2_ (Fig. 5g), which is an important driver of 20th century warming at the global scale^40^. In addition to the *p*CO_2_ forcing, Arctic warming is amplified above the global average due to reductions in sea ice concentrations, especially in the Beaufort and Chukchi Seas (north of Alaska)^41^, which likely accounts for at least part of the 20th century warming trend in our record.

### Permafrost stability and climate feedbacks

The rate and magnitude of recent climate warming has implications for the stability of ice-rich permafrost in E. Beringia, which is both widespread and sensitive to climate warming^3^. Cryostratigraphic evidence for regional failure of ice-rich permafrost in E. Beringia is linked to past hypsithermals, including thermokarst deposits in Alaska and Yukon dating to Marine Isotope Stage 5e^42^, and thaw unconformities and thaw lake initiation dating to the early Holocene in parts of Yukon and the Mackenzie Delta region^34,43–45^. However, the warming threshold for such a widespread permafrost response is not well known. Our finding that today’s summer climate (last ~30 years) exceeds the early–middle Holocene thermal maximum by +1.7 ± 0.7 °C implies that the threshold for ice-rich permafrost stability in this region has already been crossed. This finding is consistent with recent evidence of climate-driven acceleration of thaw slump activity^3,46^ and ice wedge network degradation^47^ in the Mackenzie Delta region and Western Arctic more broadly, which have been linked to climate warming. Continued warming and permafrost degradation in this region threatens to expose vast pools of frozen organic matter in near-surface permafrost to microbial activity and, if released to the global atmosphere as CO_2_ or CH_4_, has the potential to amplify future warming trends^4^.

## Methods

### Site and core collection

Ice-rich peat cores were collected from a peatland we refer to as DHP174 (65.21°N, 138.32°W; Fig. 1a–f), situated in the continuous permafrost zone. The peatland is dominated by *Sphagnum* spp. moss, and includes sparse forb and lichen groundcover, and an open canopy of *Picea mariana* trees. DHP174 is a soligenous peatland, sustained by precipitation and seasonal runoff from a south-flanking hillslope. There is ~15–20 m of relief across the ~100–150 m peatland surface, measured from the bottom of the hillslope to the northern edge of the peatland^48^. The slope ensures continuous drainage of surface and active layer runoff throughout the thaw season, and well-drained conditions in the uppermost 20–30 cm of peat. Saturated conditions during the summer occur below 20–30 cm depth and are maintained by elevated summer precipitation totals (Fig. 4d) and low saturated hydraulic conductivities (e.g. ~ 1 m day^−1^) that are typical below 30 cm depth in northern peat plateaus^27^. The DHP174 peatland sits on an alluvial terrace in a valley that drains into Engineer Creek (Fig. 1) and is underlain by permafrost, which limits runoff to the seasonally thawed active layer.

For the water isotope analysis presented in this study, we collected two 10 cm diameter core profiles using a portable permafrost drilling system^49^. The first core profile (DHP174-13, 3.83 m long) is from the ‘undisturbed’ upper surface, and the second core profile (DHP174-13L, 3.32 m long) was collected from an ‘excavated’ lower surface (Fig. 1c); the cores overlap stratigraphically, and the lower core includes the basal peats and underlying strata. The lower surface was excavated by the highway maintenance authority before 2007 (Google Earth time stamp) to thermally erode the underlying permafrost in order to expand the roadside quarry shown in the satellite images (Fig. 1d, e). At the time of coring (June 2013), frozen ground was encountered 16 and 24 cm below ground at the upper and lower coring sites, respectively, which are also the starting depths of the cores.

### Pore ice sampling and water isotope analysis

The frozen cores were split along their long axis with a bandsaw. The two core profiles were sub-sampled into 1 cm increments. For the DHP174-13 core, every tenth centimetre increment of pore ice was analysed for water isotopes starting from the core top (16 cm below the surface) to core bottom (382 cm composite depth); the only exception being DHP174-13 core depths 16–76 cm, which were analysed at near 1 cm resolution to allow for a more detailed analysis of pore ice in the active layer and across the active layer–permafrost interface. For the DHP174-13L core, every tenth centimetre increment of pore ice was analysed for water isotopes starting at a depth of 30 cm below the lower coring surface (230 cm composite depth; see Methods—chronology) down to the basal sediments at 530 cm composite depth). In total, 104 pore ice samples were analysed for water isotope ratios, 43 of which are active layer or transient layer pore ice, and 71 of which are relict pore ice.

The frozen samples were thawed at ~4 °C in plastic bags and the liquid was filtered into 2 mL autosampler vials. δD and δ^18^O ratios of the water were measured at the University of Alberta using a Picarro L2130-*i* analyser. Unknowns and standards were injected eight times, and mean raw values were calculated from the last four injections. Raw isotope ratios were corrected for inter-sample memory effects that are common to cavity ring-down spectroscopy systems^50^. We used certified water reference standards (USGS-45 and USGS-46) to normalise raw isotope ratios to the Vienna Standard Mean Ocean Water-Standard Light Antarctic Precipitation (VSMOW-SLAP) scale. Analytical error is 0.6‰ for δD and 0.1‰ for δ^18^O based on routine analysis of an internal deionised water standard.

### Peat-sediment age-depth model

A composite Bayesian age-depth model for the peat-sediment sequence was produced using radiocarbon (^14^C) dates and cryptotephra isochrons (see Davies^51^ for full details of the composite chronology). Depths are reported as core composite depths (ccd). The Bayesian age-depth model (see Supplementary Fig. 5) was constructed using the OxCal Poisson process model^52^.

Seventeen macrofossil samples were picked for ^14^C dating from the upper monolith and core profiles (Supplementary Fig. 1). This includes three dates from a previously collected core that was not used for pore ice analysis (DHP174-12, cored within 1.5 m of DHP174-13), but is included in the age model as it has precise modern cryptotephra and post-bomb ^14^C dates for its surface peat. Dates from all three cores were included in a composite profile where cryptotephra tie-points were used to correlate between the individual core profiles.

Where possible, *Sphagnum* remains were preferred as they can provide reliable ^14^C dates^53^. In the mineragenic, non-peat core sections at the base of the DHP174-13L profile, terrestrial plant macrofossils (twigs and leaves) were used for ^14^C dating. Samples were pre-treated at the University of Alberta following standard procedures^54^, and analysed for ^14^C content at the W. M. Keck Carbon Cycle AMS Laboratory (University of California, Irvine, CA, USA). Two secondary standards were pre-treated concurrently (a last interglacial non-finite-age wood, AVR-PAL-07; and a middle Holocene wood, FIRI-F) and the ^14^C results were within expected ranges. The unknown ^14^C dates were calibrated as appropriate using Bomb13NH1^55^ and IntCal13^56^ calibration curves with Oxcal v4.3.2^57^.

Glass shard concentration profiles for cryptotephra were produced at 1 cm resolution using standard methods^58^. Three samples geochemically correlated with known eruptions using major element EPMA glass analyses (Supplementary Fig. 2; see Davies^51^) were included in the composite age-depth model to improve model accuracy. Katmai-Novarupta (10 ccd) is a historic eruption with an observed occurrence date of 1912 CE. The Aniakchak CFE II (116 ccd) and Hayes set H unit F2 (133 ccd) eruptions are widely reported in Alaska and beyond, and have well-constrained Bayesian age estimates^59^.

### Chronology of relict pore ice

Meteoric waters that reach the maximum thaw depth of ~58 cm (see maximum active layer thickness or ALT_max_ in Supplementary Note 1) will eventually freeze in situ as pore ice during the fall freezeback and become ‘relict’ as permafrost aggradation occurs. Since permafrost aggradation is paced by the accumulation of materials at the surface (peat or sediment), the age of relict pore ice at any given depth below the permafrost table can be referenced from the peat-sediment age-depth model by accounting for the 58 cm offset between relict pore ice and its contemporaneous surface. For example, ca. modern relict pore ice at 58 cm composite depth is age-equivalent to the modern surface (i.e. –63 cal yr BP in the age-depth model); likewise, relict pore ice at 530 cm composite depth is age-equivalent to a paleosurface at a composite depth of 472 cm (13,585 cal yr BP in our age-depth model). We assume a static ALT_max_ of 58 cm through time. Although ALT_max_ potentially did vary throughout the Holocene due to changes in summer climate, it likely remained within 3–6 cm of the modern ALT_max_ value and does not significantly influence our pore ice chronology or conclusions (Supplementary Note 1).

### LMWL and local temperature-δ_precip_ calibration

The δ_precip_ isoscape of northern Canada is spatially complex due to regional differences in mean weather system trajectories, continentality, local orographic effects and varying degrees of continental moisture recycling^22,60^. Because each study area has its own potentially unique set of boundary conditions, δ_precip_ signals preserved in ground ice are best interpreted in the context of local δ_precip_ systematics^22^. We demonstrate the meteoric origins and seasonality of DHP174 pore ice by comparison with monthly δD and δ^18^O data^61^ from the nearest GNIP station at Mayo, Yukon, ~210 km south of DHP174 (Fig. 1a). Mayo and DHP174 are situated at similar elevations (600 and 720 m, respectively) in central Yukon in the western foothills of the Ogilvie Mountains. The Mayo data were used to constrain the LMWL (Fig. 3a), and the temperature-δ_precip_ regression lines (Fig. 1b; Supplementary Fig. 1). Three Mayo records that plotted far below the LMWL (see Fig. 3a) were excluded from the regressions because of suspected evaporative enrichment. Extreme leverage points (>3*σ* Cook’s distance) were given a weight of zero in the calibration to avoid undue bias on the regression lines. By comparison with monthly GNIP data from Whitehorse (Fig. 1a), a site that is ~325 km south of Mayo and has a similar LMWL^22^, the Mayo temperature-δ_precip_ regressions are found to be skilful in predicting δ_precip_ at other continental sites in Yukon (Supplementary Fig. 1), which supports their application to more proximal sites such as DHP174.

### Seawater correction

Precipitation isotope ratios over long timescales reflect climatic signals and changes in the isotopic composition of marine source waters (δ_seawater_). To isolate local climate signals in δ_precip (pore ice)_, which are unrelated to changes in seawater, the composite pore ice record was corrected for long-term δ_seawater_ changes following the general equation [1] from Stenni et al.^14^:

1 $${\mathrm{\delta }}_{{\mathrm{corrected}}} = {\mathrm{\delta }}_{{\mathrm{ice}}} - {\mathrm{\delta }}_{{\mathrm{seawater}}} \times \left( {{\mathrm{1}} + {\mathrm{\delta }}_{{\mathrm{ice}}}{\mathrm{/1000}}} \right){\mathrm{/}}\left( {{\mathrm{1}} + {\mathrm{\delta }}_{{\mathrm{seawater}}}{\mathrm{/1000}}} \right).$$

We assume the δ^18^O_seawater_ reconstruction by Rohling et al.^62^ and that δD_seawater_ changes were ~8× larger than δ^18^O_seawater_, as supported by data from Late Pleistocene marine pore waters^63^ and as assumed by others^14^.

### Temperature reconstruction

Two types of temperature estimates were made based on the pore ice record—mean temperatures for the modern era (Fig. 4c) and a full Holocene reconstruction of temperature anomalies (Δ*T*) (Fig. 5d). The first was calculated based on δD_pore ice_ and δ^18^O_pore ice_ of ca. modern relict pore ice (58–61 cm, uppermost 3 cm of permafrost from the DHP174-13 core), and was used as a line of evidence to validate the proposed summer seasonality of pore ice. Mean temperature estimates depend on the slope (*m*) and intercept (*b*) of the δ_precip_-temperature regression line, and δ_pore ice_ values corrected for the first ice enrichment effect (ε_ice-water_; 5.8‰ and 0.8‰ for δD_pore ice_ and δ^18^O_pore ice_, respectively; Supplementary Note 2). Uncertainties in the mean temperature estimates take into account the 1*σ* error in δD_pore ice_ and δ^18^O_pore ice_ measurements, and 1*σ* uncertainties in the regression slope and intercept. The general formula used to calculate mean temperatures based on either isotope is: *T* = ([δ_pore ice_ ± 1*σ*] –  *ε*_ice-water_ – [*b* ± 1*σ*])/[*m* ± 1*σ*].

Temperature anomalies (Δ*T*) were reconstructed for the full Holocene based on δD_pore ice_ anomalies (ΔδD_pore ice_, relative to Holocene mean) and the δD_precip_–temperature slope (*m*). Since ε_ice-water_ is assumed to be a constant (Supplementary Note 2), ΔδD_pore ice_ and ΔδD_precip_ are equal. The formula used for estimating Δ*T* is: Δ*T* = [ΔδD_pore ice_ ± 1*σ*]/[*m* ± 1*σ*].

## Supplementary information

Supplementary Information

Supplementary Data 1

## Data Availability

The DHP174 isotope data and reconstruction are provided in the online supplement.
