# Supplementary material for: Recent summer warming in northwestern Canada exceeds the Holocene thermal maximum
Source: Nat Commun. 2019 Apr 9;10:1631. doi: 10.1038/s41467-019-09622-y (PMC6456611; doi:10.1038/s41467-019-09622-y)
Supplement: Supplementary file 1 — Supplementary Information [file 41467_2019_9622_MOESM1_ESM.docx]

**SUPPORTING INFORMATION**

**Recent summer warming in central Yukon exceeds the Holocene thermal maximum**

Trevor J. Porter^1,*^, Spruce W. Schoenemann^2^, Lauren J. Davies^3^, Eric J. Steig^4^, Sasiri Bandara^3^, Duane Froese^3^

^1^Department of Geography, University of Toronto, Erindale Campus, Mississauga, ON, Canada

^2^Environmental Sciences Department, University of Montana Western, Dillon, MT, USA

^3^Department of Earth and Atmospheric Sciences, University of Alberta, Edmonton, AB, Canada

^4^Department of Earth and Space Sciences, University of Washington, Seattle, WA, USA

*Corresponding author: Trevor Porter (trevor.porter@utoronto.ca)

**Supplementary Note 1.** Maximum active layer thickness (ALT_max_)

ALT_max_ is the maximum depth to which meteoric waters can percolate in the summer thaw season, and where pore ice becomes relict if the permafrost table subsequently aggrades above that position (paced by the accumulation of sediment or peat at the surface). It is also a key parameter for deriving the chronology of relict pore ice from the peat-sediment age-depth model (see Methods). In this section we constrain the ALT_max_ parameter for the DHP174 site based on Active Layer Thickness (ALT) measurements from the 2013 and 2016 thaw seasons, the latter being one of the warmest and wettest thaw seasons according to local climate records, and preceded by an especially warm winter-spring owing to the 2015-16 El Niño. As ALT is sensitive to summer warmth^1^, the 2016 measurements offer particularly useful constraints on ALT_max_.

At the time of coring (June 19, 2013), the thaw depth was 16 cm at the upper coring site (DHP174-13) and 24 cm at the lower coring site (DHP174-13L). The deeper thaw at the lower site on the same day is due, in part, to the legacy of disturbance of the lower surface (caused by the excavation; see Methods – Site and core collection) which today has a relatively low albedo due to decomposed peats and poor drainage. Prior to the disturbance, the two coring sites likely had similar thaw depths given their close proximity and the uniform vegetation cover (primarily peat moss) through time. In 2016, mean ALT values of 47 cm and 48 cm were measured by frost probe at two locations next to the upper coring site on August 2, and duplicate measurements of 56 cm from the same two locations on September 13 near the end of the thaw season.

Monitoring studies in Alaska^2^ and Northwest Territories^1^ demonstrate that ALT can be skillfully modelled as a linear function of the squared root of the cumulative Thawing Degree Day index (TDD^1/2^; TDD = sum of mean daily air temperatures > 0°C). TDD was estimated for DHP174 site for the days corresponding to the 2013 and 2016 ALT measurements. We used the daily ERA-Interim 2 m temperature product^3^, which agrees (r = 0.99, p < 0.01) with the available daily temperature record from the nearby Ogilvie River climate station (1971-2007; Supplementary Note 3). A linear ALT-TDD^1/2^ response also aptly describes the 2013 and 2016 ALT data (Supplementary Figure 6). A line-of-best fit constrained by the 2016 (late thaw season) measurements has a slope of 1.56 cm per TDD^1/2^ with its intercept set to zero (i.e., ALT = 0 cm at the start of the thaw season). This slope is within range of ALT-TDD^1/2^ slopes observed by Smith et al.^1^ for Arctic taiga peatlands in the Mackenzie Valley. Our 2013 early thaw season ALT data also plot close to this line but are associated with larger residuals, which is typical for other sites in the early thaw season^1,2^.

Based on the ALT-TDD^1/2^ line-of-best fit (Supplementary Figure 6), it is also possible to predict the end-of-season ALT for 2016 given an end-of-season TTD^1/2^ estimate. According to the ERA-Interim record, the last thaw day (i.e., mean daily temperatures above 0°C) was September 26 (13 days after the last frost probe measurement). The TDD gain from September 13-26 was ~57°C, which equates to an additional ~1 cm of thaw and a total ALT of 57.5 cm for the 2016 thaw season.

Finally, we also calculated TDD and ALT for the DHP174 site for each year of the ERA record (1979-2016). The 2016 ALT estimate exceeds that of all other years, but is similar to ALT estimates for 1989, 2010 and 2013 which range from 56.3-56.4 cm. For the 1979-2016 period, ALT estimates range from 47.3 cm during cooler-than-average summers to a maximum of 57.5 cm. While the active layer is unlikely to thaw to ALT_max_ during most years, it is the infrequent, deeper-than-normal thaw events (e.g., 1989, 2010, 2013) that can potentially reanimate and mix with pore ice that froze at intermediate thaw depths during previous, cooler summers. As such, even meteoric waters from cooler-than-normal summers are likely to be represented in pore ice at ALT_max_.

Because the 2016 thaw season was likely the warmest on record, we contend that ~58 cm is a reasonable approximation for ALT_max_ under the modern climate regime. For deriving the full Holocene chronology of relict pore ice (see Methods – Pore ice chronology), we assume ALT_max_ was constant. This assumption would be true under an unchanging climate, but major changes in mean summer temperatures (and TDD^1/2^) could undermine that assumption. However, under the range of reconstructed summer temperatures for this region (Figure 5d), ALT_max_ likely did not vary much from the modern value. Today (and likely in the past), warmer summers equate to a higher TDD^1/2^ index. According to the ERA-Interim record, TDD^1/2^ increases by 1.2 units for every 1°C increase in average July-August temperatures (r = 0.93, p < 0.01); given that sensitivity and the ALT-TDD^1/2^ response (Supplementary Figure 6), the ALT-temperature sensitivity is estimated to be 1.9 cm·°C^-1^ (1.2 TDD^1/2^·°C^-1^ × 1.56 cm·TDD^-1/2^), which is consistent with model-based sensitivity estimates of ALT for thick Organic-layer (>1 m) soils in NW Canada^4^. Given this sensitivity and the range of reconstructed summer temperature anomalies for this site (within ~1.5-3°C of modern; Figure 5d), ALT_max_ likely remained within 3-6 cm of the modern value during the Holocene. This has minor implications for the pore ice chronology. Considering the average peat accumulation rate of ~0.4 cm/decade and ~3-6 cm of ALT_max_ variability, the relict pore ice chronology can be considered accurate to within 75-150 years of the modelled age. Since we focus on centennial to millennial-scale trends in paleoclimate, 75-150 years of chronological error is inconsequential to our results and conclusions.

**Supplementary Note 2.** Fractionation of active layer pore ice

Water isotopes are fractionated during active layer freezing, with a preference for heavy isotopologues in the solid phase^5^. In permafrost regions, active layer freezeback is generally bi-directional (top-down and bottom-up) since excess heat from the warm active layer dissipates to the colder atmosphere and permafrost in winter. The first pore ice to form (hereafter ‘first ice’), which occurs at the upper and lower freezing fronts, is expected to be most enriched in heavy isotopes compared to the residual, unfrozen pore waters. Because this fractionation leaves the residual waters depleted in heavy isotopes, subsequent pore ice will always be less enriched than first ice^5^. In natural active layers where freezing is bi-directional, enriched first ice is expected at the top and bottom of the active layer, and the most depleted ‘last ice’ in the middle active layer where the freezing fronts converge. This pattern is observed in DHP174-13 active layer pore ice profile (Supplementary Figure 7).

The DHP174-13 active layer shows the most enriched pore ice at a depth of 47 cm (δD = –153.6 ‰, δ^18^O = –19.2 ‰) and the most depleted pore ice in the middle active layer at 26 cm (δD = –168 ‰, δ^18^O = –21.3 ‰) (Supplementary Figure 7; n.b., pore ice above 16 cm depth was not recovered at the time of coring since the ground had already thawed above this depth). The most enriched first ice at 47 cm likely marks the maximum thaw depth of the 2012 active layer (i.e., before the year of coring). Pore ice situated between the maximum active layer thickness (ALT_max_; Supplementary Note 1) and the 2012 active layer thickness (ALT_2012_), a zone of intermediate thaw depths (referred to as the ‘transient layer’ in the permafrost literature), represents pore ice that formed in prior thaw seasons.

The magnitude of the first ice enrichment over initial water (ε_ice-water_) must be constrained to better understand the precipitation seasonality of relict pore ice. The value of ε_ice-water_ depends on the rates of freezing and isotopologue diffusion^5^. Under equilibrium (or slow) freezing when isotopic diffusion exceeds the freezing rate, ε_ice-water_ values of ~20 ‰ for δD and 3 ‰ for δ^18^O are theoretically possible^5^, but such values are largely unfounded in natural active layer studies (e.g., Lacelle et al.^6^). The total ranges of δD and δ^18^O observed in the DHP174-13 active layer are 14.4 ‰ and 2.1 ‰, respectively. Since these ranges represent the full distillation series of first to last ice, the enrichment of first ice above the initial source water must be even smaller. Conservation of mass dictates that initial pore water δD and δ^18^O values must be intermediate between first ice and last ice δD and δ^18^O. The arithmetic mean of pore ice δD and δ^18^O values from the series of 2012 active layer pore ice can be used to approximate the initial pore water δD and δ^18^O, which we use to estimate ε_ice-water_. Accordingly, we estimate that first ice is enriched above initial water by ~5.8 ‰ for δD and ~0.8 ‰ for δ^18^O. These enrichments are less than a third of ε_ice-water_ values that are typically observed for pure water systems at 0°C^7^. However, pure water systems are a poor analogue for natural soils with solute-rich pore waters that freeze at sub-zero temperatures, and complex hydrological pathways (e.g., collapsed and dead-end pore space in peats^8^) which could potentially inhibit molecular diffusion and, thus, isotopologue enrichments.

Variability in ε_ice-water_ through time is also a significant uncertainty with implications for interpretation of climate signals from long-term trends in pore ice δD and δ^18^O. Changes in ε_ice-water_ might occur due to changes in sedimentary boundary conditions (e.g., peat vs. minerogenic soil) or climate-driven effects on ALT, which might be expected to impact the rates of freezing and diffusion. However, the DHP174-13 active layer pore ice offers some evidence that ε_ice-water_ does not vary greatly under a variable ALT. We note that ‘first ice’ at ALT_2012_ (δD = –153.6 ‰, δ^18^O = –19.2 ‰) is isotopically consistent with pore ice found at ALT_max_ (δD = –154.5 ‰, δ^18^O = –19.4 ‰) which likely represents first ice that formed during a previous albeit unknown thaw season. This similarity is in spite of ALT_2012_ being ~10 cm shallower than ALT_max_, and implies that ε_ice-water_ does not vary much between warm and cool summers. The molecular diffusion rate at the lower freezing front can also be considered a constant through the Holocene portion of our record due to the uniform peat moss lithology, and saturated conditions below ~20 cm. Further, owing to saturated conditions, the rate of freezing is also expected to be relatively constant. Heat propagation and freeze-up in a saturated active layer is dominated by latent heat effects. As the saturated active layer loses heat to atmosphere above and to permafrost below, it will approach an isothermal state at the freezing point, a phenomenon known as the ‘zero curtain effect’^9^. At this point, the freezing rate at the upper and lower freezing fronts is thermally buffered by latent heat released from freezing water, which ensures a slow, controlled rate of freezing, and offers a reasonable mechanism to explain complacency in ε_ice-water_.

The sensitivity of ε_ice-water_ to boundary conditions in natural active layer settings is not well documented in the literature. However, relict pore ice-based reconstructions would benefit from more research on this topic given its potential influence on δ_pore ice_ signals, especially where boundary conditions (e.g., surface vegetation, lithology or water table) have varied significantly in the past. For the purposes of our reconstruction, we assume that ε_ice-water_ was stable throughout the Holocene portion of the record, which is defined by a constant peat lithology since 11.5 ka BP and likely had similar hydraulic and thermal properties since peatland inception. However, the Pleistocene portion of our record is associated with clastic and biogenic-clastic lithologies, and the implications of different sedimentological boundary conditions for ε_ice-water_ is unknown. Thus, isotopic trends in the Pleistocene portion of the record should be interpreted with caution.

**Supplementary Note 3.** Local climate data

Monthly precipitation totals and mean monthly temperatures from the Ogilvie River climate station (1971-2007; Station ID 2100794; available at: <http://climate.weather.gc.ca/>), ~17 km north of the DHP174 site, were used to calculate the modern climatology of the study area. Temperature and precipitation data from the Dawson City Airport Environment Canada station (Station ID 2100402; available at: <http://climate.weather.gc.ca/>) were used to discuss conditions during the warm and wet 2016 thaw season.

**Supplementary Table 1**. Summary of ^14^C dates produced from the three peat cores.

| **Sample ID** | **UCIAMS #** | **Depth (cm)** | **Depth (ccd)** | **Material dated** | **Fraction modern** | **±** | **^14^C age (BP)** | **±** |
| --- | --- | --- | --- | --- | --- | --- | --- | --- |
| DHP174-12 | 167484 | 4 | 4 | *Sphagnum* stems | 1.208 | 0.0022 | --- | --- |
|  | 167482 | 14 | 14 | *Sphagnum* stems | --- | --- | 165 | 20 |
|  | 114734 | 64 | 64 | *Sphagnum* stems | --- | --- | 2190 | 25 |
| DHP174-13 | 142067 | 22 | 22 | *Sphagnum* stems | --- | --- | 1165 | 45 |
|  | 131060 | 260 | 260 |  | --- | --- | 6445 | 25 |
|  | 142072 | 368 | 368 | *Sphagnum* stems | --- | --- | 8610 | 30 |
|  | 131061 | 385 | 385 | *Sphagnum* stems | --- | --- | 8670 | 120 |
| DHP174-13L | 131062 | 25 | 215 | *Sphagnum* stems | --- | --- | 5380 | 25 |
|  | 142071 | 106 | 296 | *Sphagnum* stems | --- | --- | 7435 | 30 |
|  | 142069 | 186 | 376 | *Sphagnum* stems | --- | --- | 8780 | 70 |
|  | 142070 | 214 | 404 | *Sphagnum* stems | --- | --- | 9290 | 25 |
|  | 131063 | 244 | 434 | Woody fragments | --- | --- | 9265 | 40 |
|  | 167483 | 278 | 468 | Graminoid macrofossil | --- | --- | 12305 | 30 |
|  | 131068 | 331 | 521 | Graminoid macrofossil | --- | --- | 13040 | 240 |

**Supplementary Table 2**. Cryptotephra correlations included in the composite age-depth model*.*

| **ID** | **Depth (ccd)** | **Correlated eruption** | **Eruption age** |
| --- | --- | --- | --- |
| DHP174-12 | 10 | Novarupta-Katmai 1912 | 1912 CE |
| DHP174-13 | 116 | Aniakchak CFE II | 3475±80 cal yr BP |
| DHP174-13 | 133 | Hayes set H unit F2 | 4075±150 cal yr BP |


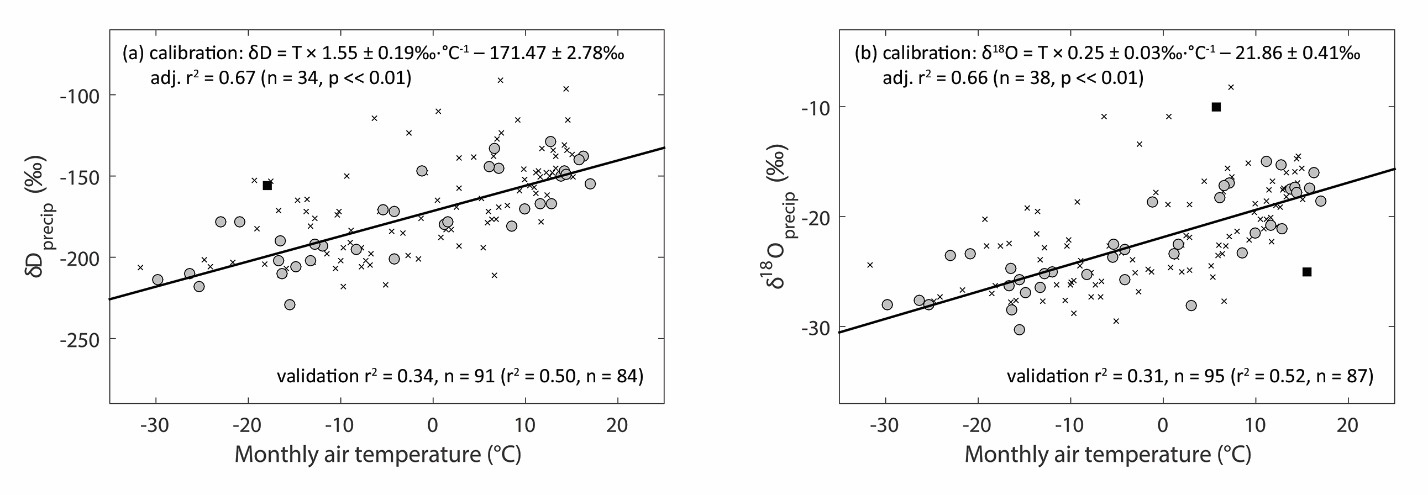


**Supplementary Figure 1.** (a) δD_precip_-temperature and (b) δ^18^O_precip_-temperature regression lines calibrated with Mayo GNIP data (circles; excluding any leverage points marked with a square, see Methods); the validation r^2^ reflects the performance of the calibration models in predicting Whitehorse GNIP δD_precip_ and δ^18^O_precip_ (crosses); the validation r^2^ in brackets excludes extreme outlier residuals >2.5× the interquartile range.


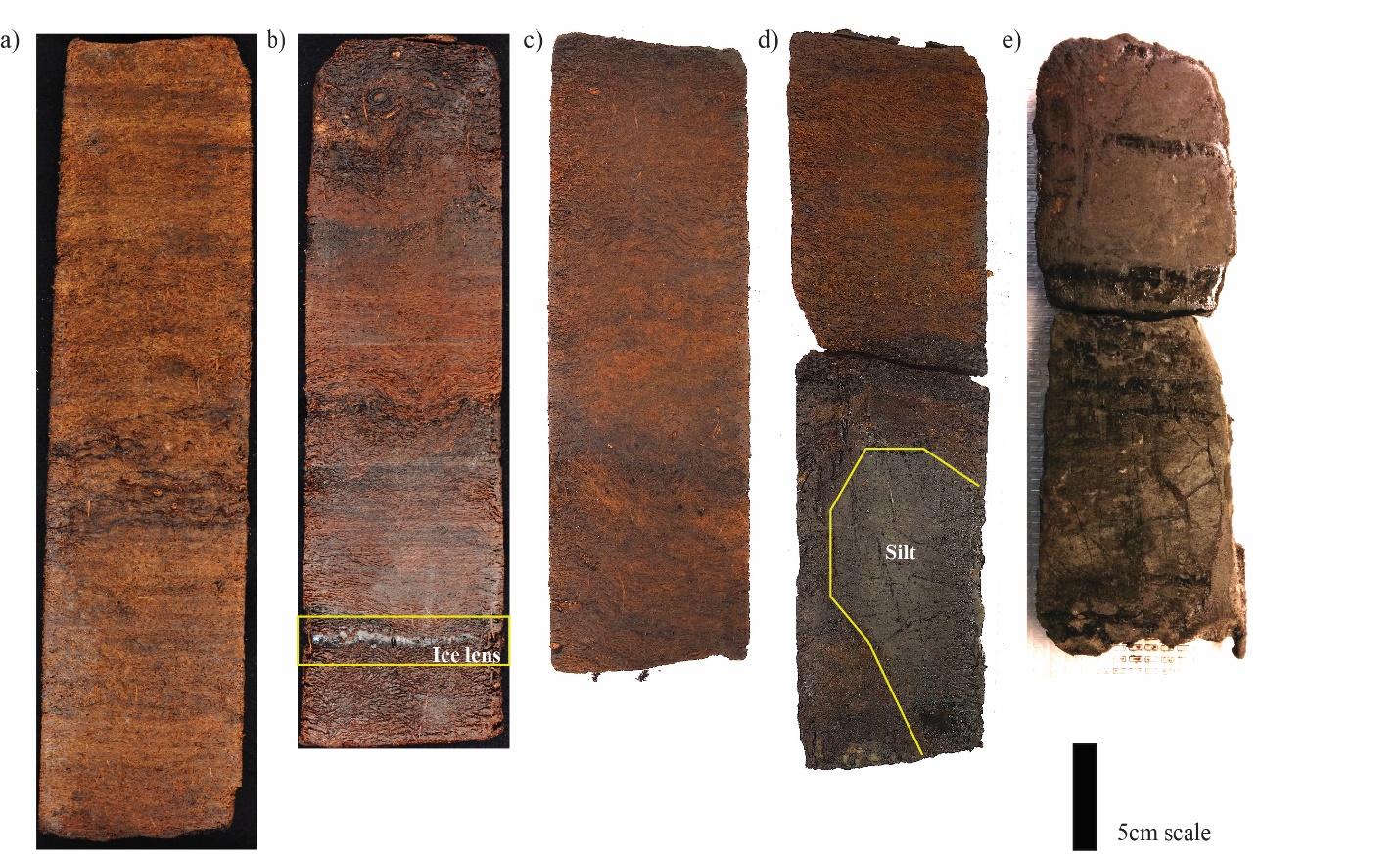


**Supplementary Figure 2.** Photos of frozen core slices from DHP-174. Typical Holocene peat material from the upper core profile DHP174-13 (a, b) and lower core profile DHP174-13L (c); one of the rare ice lenses observed in the peat unit is shown in (b). The silt-peat transition in DHP174-13L (d). Pre-Holocene silt in DHP174-13L (e).


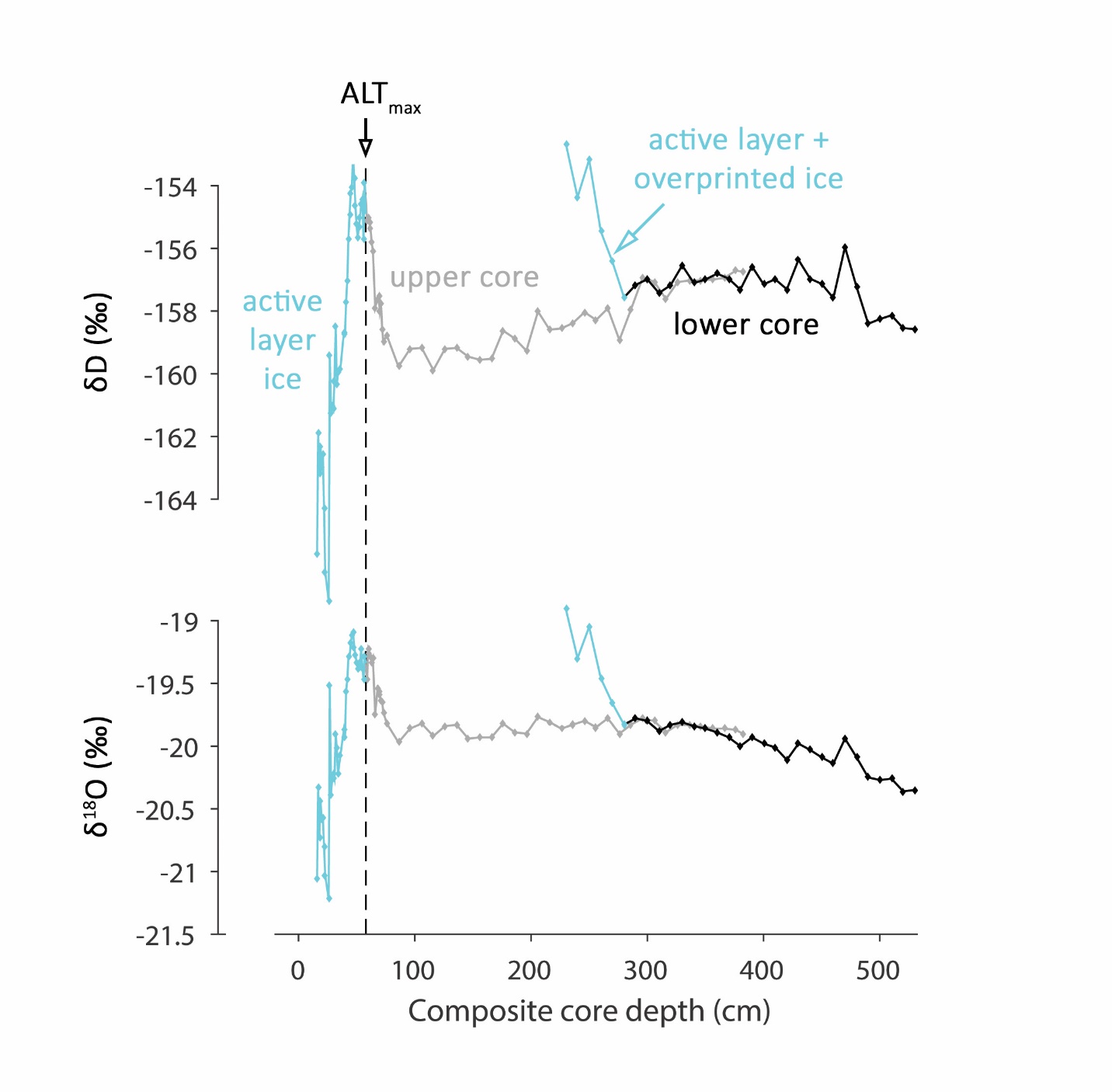


**Supplementary Figure 3.** A comparison of δD_pore ice_ and δ^18^O_pore ice_ series (offset-adjusted; as in Figure 2a-b) plotted against composite depth. Relict pore ice is indicated for the upper (grey) and lower core (black). Upper core active layer pore ice (16-57 cm), and lower core ‘overprinted’ and active layer pore ice (230-280 cm) are indicated (cyan line). The point at which lower core δ_pore ice_ diverges from the upper core relict pore ice δ_pore ice_ series (composite depths < 290 cm depth; or <90 cm below the lower coring surface) marks the depth to which lower core pore ice has likely been overprinted by modern pore waters related to the site disturbance prior to 2007 (see Methods – site info).


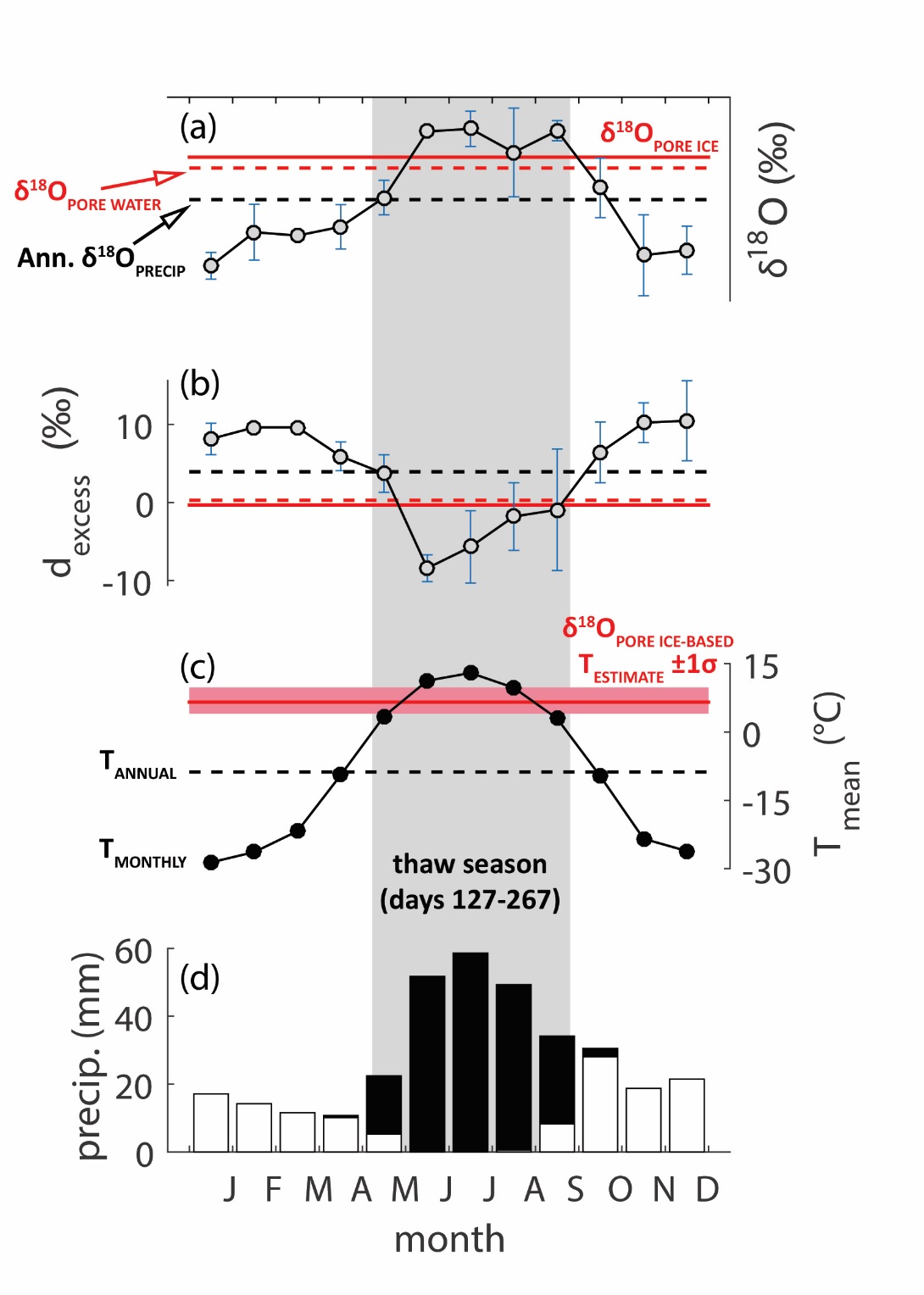


**Supplementary Figure 4.** Precipitation and pore ice isotope climatology. Mean monthly **(a)** δ^18^O_precip_ and **(c)** *d*_excess_ calculated from Mayo GNIP data (grey circles and 1σ error bars; excludes suspected evaporation enriched data points from Figure 3a); also shown are mean annual δ^18^O_precip_ and *d*_excess_ (dashed black lines), and DHP174-13 (top-of-permafrost, 58-60 cm) δ^18^O_pore ice_ (solid red line) and initial δ^18^O_pore water_ (dashed red line); **(c)** comparison of a δ^18^O_pore ice (top-of-permafrost)_-based temperature estimate (red line; red band indicates the 1σ uncertainty in the estimate; Methods) and observed mean monthly temperatures at the Ogilvie River climate station; **(d)** average total monthly snowfall (white bars; snow water equivalent) and rain (black bars) at Ogilvie River.


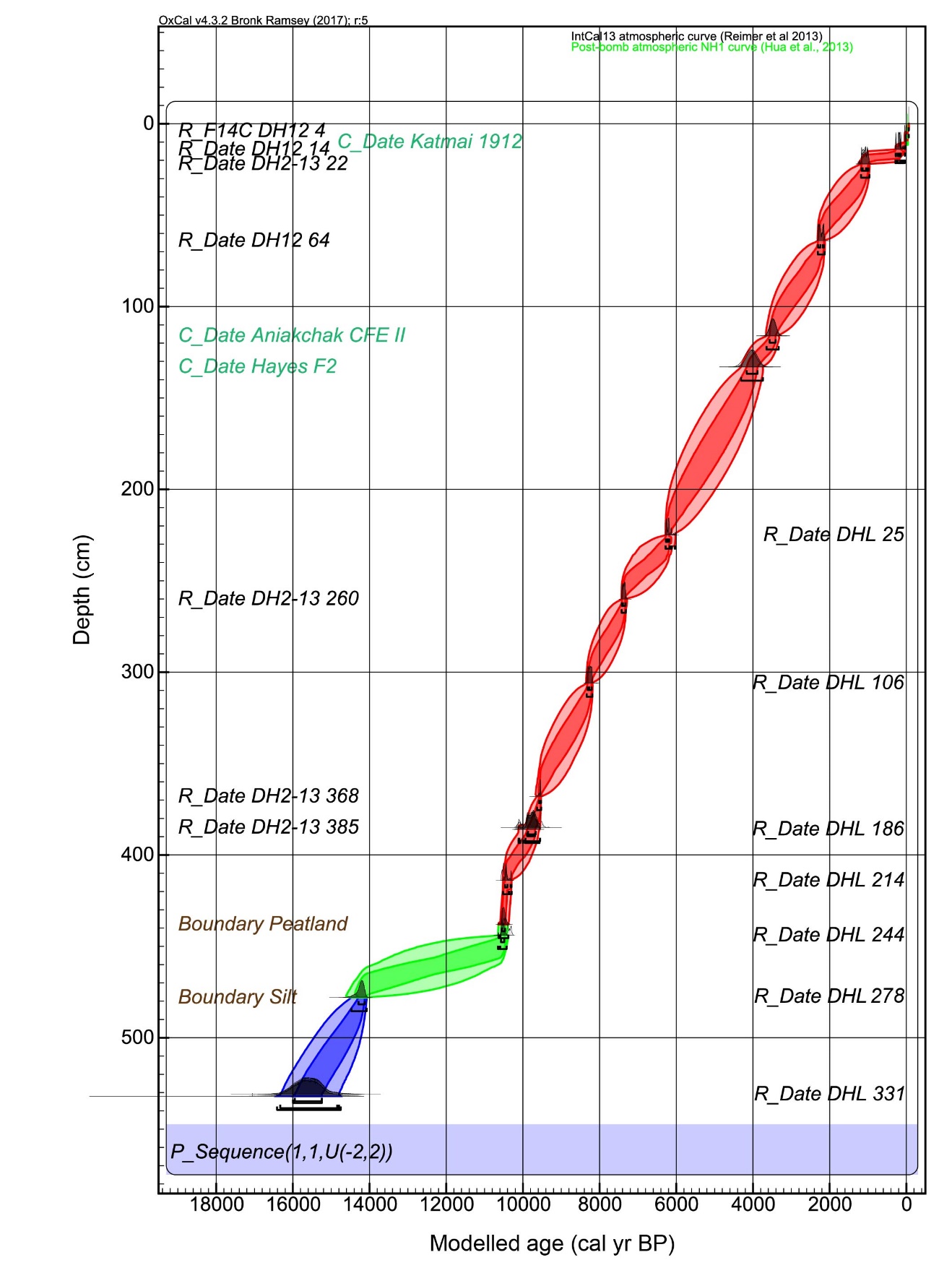


**Supplementary Figure 5**. Composite Bayesian age-depth model for DHP174 produced using OxCal v4.3.2. Dates are offset or highlighted in colour for clarity. R_Date = ^14^C date; R_F14C = post-bomb ^14^C date; C_Date (green) are used here for cryptotephra isochrons.


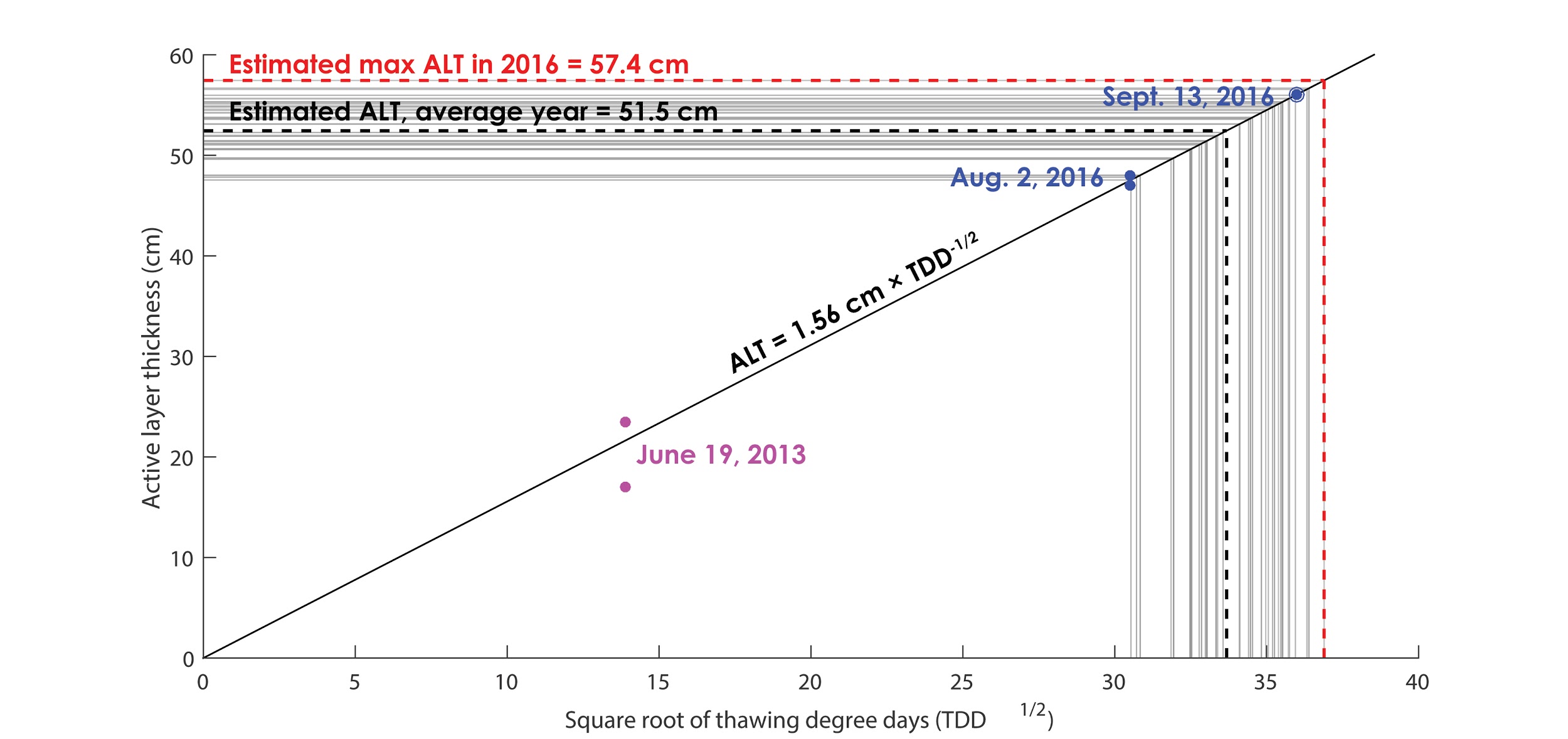


**Supplementary Figure 6.** Measured active layer thickness (dots) at the DHP174 site versus the squared root of the Thawing Degree Day index (TDD^1/2^); the TDD was estimated for the days corresponding to active layer measurements using the ERA-Interim 2 m daily mean air temperature product^3^. The 2016 ALT observations and 0 cm intercept define the line-of-best fit (ALT = 1.56 cm × TDD^-1/2^). Using this line and (ERA-interim-based) TDD estimates, end-of-thaw-season (or total) ALT was estimated for all years from 1979-2016 (grey lines) and the average year (black dashed); ALT_max_ (red dashed line) is also indicated.


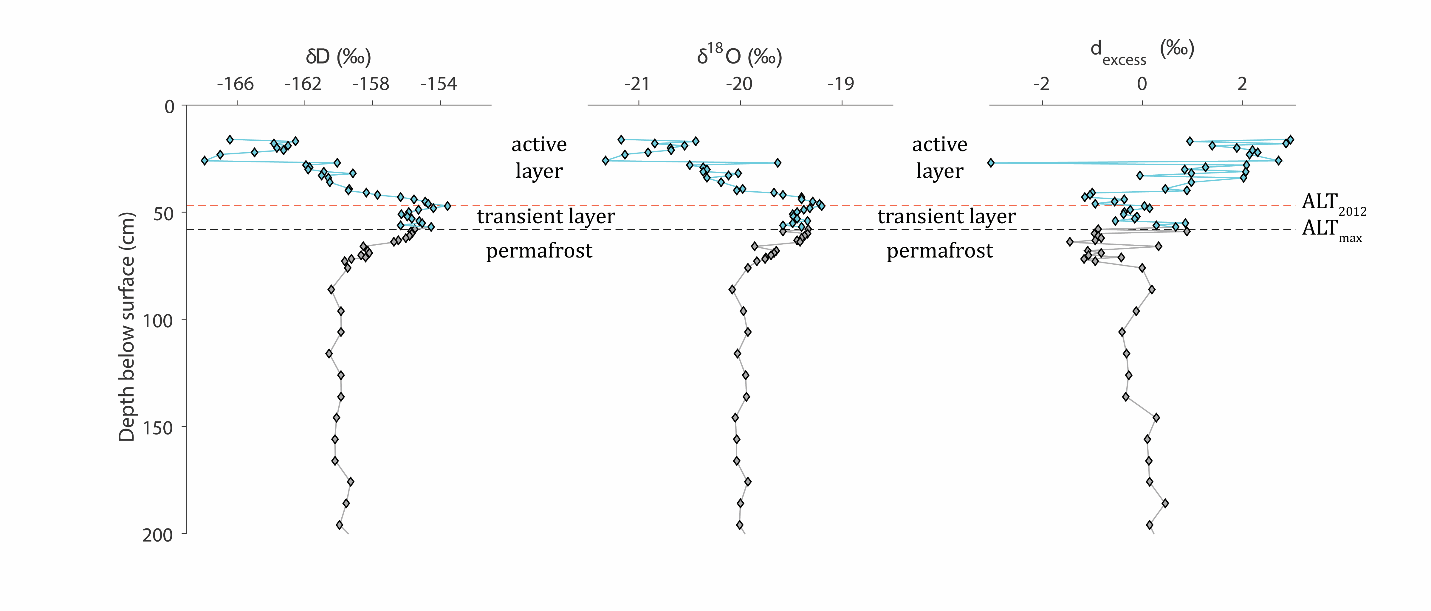


**Supplementary Figure 7.** Near surface pore ice δD_,_ δ^18^O and *d*-excess profiles for the DHP174-13 core (upper site); ALT_max_ (58 cm) and ALT for the 2012 thaw season (47 cm) are marked with dashed lines.

**References**

1. Smith, S. L., Wolfe, S. A., Riseborough, D. W. & Nixon, F. M. Active-Layer Characteristics and Summer Climatic Indices, Mackenzie Valley, Northwest Territories, Canada. *Permafr. Periglac. Process.* **20,** 201–220 (2009).

2. Romanovsky, V. E. & Osterkamp, T. . Thawing of the Active Layer on the Coastal Plain of the Alaskan Arctic. *Permafr. Periglac. Process.* **8,** 1–22 (1997).

3. Dee, D. P. *et al.* The ERA-Interim reanalysis: configuration and performance of the data assimilation system. *Q. J. R. Meteorol. Soc.* **137,** 553–597 (2011).

4. Woo, M., Mollinga, M. & Smith, S. L. Climate warming and active layer thaw in the boreal and tundra environments of the Mackenzie Valley. *Can. J. Earth Sci.* **44,** 733–743 (2007).

5. Lacelle, D. On the δ18O, δD and d-excess relations in meteoric precipitation and during equilibrium freezing: theoretical approach and field examples. *Permafr. Periglac. Process.* **22,** 13–25 (2011).

6. Lacelle, D., Fontaine, M., Forest, A. P. & Kokelj, S. High-resolution stable water isotopes as tracers of thaw unconformities in permafrost: A case study from western Arctic Canada. *Chem. Geol.* **368,** 85–96 (2014).

7. Suzuoki, T. & Kumura, T. D/H and 18O/16O fractionation in ice-water systems. *Mass Spectrosc.* **21,** 229–233 (1973).

8. Rezanezhad, F. *et al.* Structure of peat soils and implications for water storage, flow and solute transport: A review update for geochemists. *Chem. Geol.* **429,** 75–84 (2016).

9. Outcalt, S. I., Nelson, F. E. & Hinkel, K. M. The zero-curtain effect: Heat and mass transfer across an isothermal region in freezing soil. *Water Resour. Res.* **26,** 1509–1516 (1990).
